# The need for synergy between biological and behavioral approaches to address accelerated weight gain during the summer in children

**DOI:** 10.1186/s12966-019-0800-y

**Published:** 2019-04-29

**Authors:** Michael W. Beets, Keith Brazendale, R. Glenn Weaver

**Affiliations:** Arnold School of Public Health, University of South Carolina, Columbia, USA

**Keywords:** Childhood obesity intervention structure

We would like to recognize the important contribution from Moreno and colleagues [1] regarding their proposed biological mechanisms that underpin changes in seasonal weight gain among youth and how these may play a role in the phenomenon of accelerated weight gain during summer. In their article, Moreno et al. present a compelling argument regarding the role of biology, specifically circadian and circannual rhythms (referred to as the circadian and circannual rhythm model, CCRM), in conjunction with energy balance behaviors that are influenced by “social demands”, in seasonal differences in growth patterns of height and weight of youth.

The biological focus of the CCRM proposes children display seasonal rhythmicity in height and weight growth patterns, synchronized by exposure to natural light-dark cycles that differ by season. Moreno et al. present evidence suggesting children are more prone to gain weight during summer, compared to winter, leading to the accelerated weight gain observed across studies [2–8]. This heightened biological susceptibility over summer to gain weight, when coupled with alterations in “social demands”, are hypothesized to lead children to exhibit greater variability in traditional energy balance behaviors (i.e., physical activity, diet, sleep and screen time). These two changes, one biological and the other behavioral, are hypothesized to lead to greater gains in weight and, subsequently, overweight and obesity prevalence in children.

We believe the novel biological perspective within the CCRM helps further the field’s understanding of how and when seasonal changes in height and weight may be biologically primed and lead to excessive adiposity (i.e., accelerated weight gain) – a phenomenon that has a robust literature base from numerous studies dating from the 1990s to the present day [2–8].

However, we would like to comment on the implications of CCRM to inform strategies to prevent and treat childhood obesity during the summer. We argue that while the CCRM provides a novel biological explanation for why accelerated summer weight gain may be occurring, the interventions for addressing summer weight gain are predominately behavioral and, apart from light therapy, indistinguishable from those advocated by the Structured Days Hypothesis (SDH) [9].

Moreno et al. suggest changes in “social demands” facilitate circadian misalignment through changes in traditional energy balance behaviors during specific times of the year, ultimately leading to accelerated weight gain. “Social demands” are defined as work/school schedules, community involvement, family routines, and/or parenting practices, and are suggested as mechanisms that can lead to disruptions and variability in the timing of sleep, meals, and activity patterns of children. These “social demands” are different during the summer, compared to the school year, and result in less favorable energy balance behaviors.

We agree that summer vacation changes children’s “social demands” which can lead to less favorable energy balance behaviors. However, the solutions Moreno and colleagues propose for addressing the impact “social demands” may have on energy balance behaviors predominately focus on elements outlined within the SDH. Explicitly detailed in the SDH is the role that structure, and its presence or absence, may play in mitigating or promoting accelerated weight gain. The SDH proposes a comprehensive model towards understanding the physical activity, diet, screen time/sedentary, and sleep behaviors of children and how these behaviors are beneficially regulated by the introduction of structure during the school year. With the removal or lessening of structure over the summer, the SDH theorizes children exhibit less healthful behaviors during this time period.

In absence of studies examining within child differences in energy balance behaviors between school and summer, the SDH was grounded in a large number of international studies that compared days when children were exposed to more structure (i.e., school week day) versus days with potentially less structure (i.e., weekend days). Across all four energy balance behaviors (i.e., physical activity, sleep, diet, screen time), ~ 80% of 190 studies demonstrated children exhibited more favorable energy balance behaviors while in school compared to weekends. This led to the question of whether summer could be theoretically viewed as one long weekend.

In further support of the SDH, studies are now emerging that provide initial evidence that when youth are experimentally exposed to some form of regularly scheduled, routine-driven structured programming during summer, improvements in their traditional energy balance behaviors, as well as maintenance or improvements in their weight status, occur [10–14]. Importantly, these studies indicate structured programming of any kind, not just programming that focuses explicitly on the more traditional energy balance behaviors of physical activity and diet, mitigate the acceleration of weight during summer.

We believe structured programming does this from the presence of compulsory, restrictive, and regulated components. As outlined in the SDH, these components include:

- The consistency of bed/wake times to meet the scheduled demands of attending a program (or school) on a subsequent morning;
- Regulating dietary intake through provided meals that are calorically capped, consistent in timing, and limited in number;
- Limiting access to screen time and the co-occurrence of snacking during sedentary behaviors; and
- Promoting compulsory physical activity, either intentionally through designated times, such as outdoor play time or unintentionally through transitions between locations of activities

Although larger-scale, controlled trials are needed, these studies [10–14] are significant because they indicate that regularly scheduled, routinized structured programming during summer influences energy balance behaviors and weight gain. These studies are also important because the programming mimics many of the features which typically occur during school, which is a period of time that shows a beneficial impact on child weight trajectories [2–8].

Whether behavioral approaches utilize strategies referred to as “structure”, as within the SDH, or as Moreno et al. define as “schedules,” “routines” or “social demands” is not important. What is important is the presence (or absence) of structure during a time of the year where children are biologically primed for weight gain. Taken together, the accumulating evidence indicates that some form of structure, whether outside the home, as in community-based programs, or within the home from consistent parenting rules/routines, are key intervention elements for obesity prevention or treatment during this time period.

Thus, providing structure during summer when children are biologically more susceptible to accelerated weight gain may be beneficial to addressing childhood obesity. It is our position that the biological perspective of the CCRM presented by Moreno and colleagues helps to explain why structure, as outlined in the SDH, may be beneficial. We believe these two complementary models, one describing the biological (CCRM) and the other the behavioral (SDH) perspectives, can together produce advancements in our understanding of the etiology of excessive weight gain and enhance our ability to develop more impactful public health interventions.

## Abbreviations

CCRM: Circadian and circannual rhythm model
SDH: Structured Days Hypothesis
